# Accelerating vaccine development and deployment: report of a Royal Society satellite meeting

**DOI:** 10.1098/rstb.2011.0100

**Published:** 2011-10-12

**Authors:** Migena Bregu, Simon J. Draper, Adrian V. S. Hill, Brian M. Greenwood

**Affiliations:** 1 Jenner Institute, University of Oxford, Oxford, UK; 2 Department of Infectious and Tropical Diseases, London School of Hygiene and Tropical Medicine, London, UK

**Keywords:** human vaccines, veterinary vaccines, vaccine development, vaccine implementation

## Abstract

The Royal Society convened a meeting on the 17th and 18th November 2010 to review the current ways in which vaccines are developed and deployed, and to make recommendations as to how each of these processes might be accelerated. The meeting brought together academics, industry representatives, research sponsors, regulators, government advisors and representatives of international public health agencies from a broad geographical background. Discussions were held under Chatham House rules. High-throughput screening of new vaccine antigens and candidates was seen as a driving force for vaccine discovery. Multi-stakeholder, small-scale manufacturing facilities capable of rapid production of clinical grade vaccines are currently too few and need to be expanded. In both the human and veterinary areas, there is a need for tiered regulatory standards, differentially tailored for experimental and commercial vaccines, to allow accelerated vaccine efficacy testing. Improved cross-fertilization of knowledge between industry and academia, and between human and veterinary vaccine developers, could lead to more rapid application of promising approaches and technologies to new product development. Identification of best-practices and development of checklists for product development plans and implementation programmes were seen as low-cost opportunities to shorten the timeline for vaccine progression from the laboratory bench to the people who need it.

## 1. Introduction

Vaccines are the greatest contribution modern medicine has made to humanity, providing a powerful and cost-effective intervention to prevent deadly diseases. Successful immunization campaigns have achieved eradication of smallpox and rinderpest (a viral disease of cattle) and poliomyelitis is close to worldwide elimination. In spite of these strides, infectious diseases still kill millions of people every year, with developing countries remaining particularly vulnerable. The development of a human vaccine from concept to licensure currently takes at least 15 years, while introduction plans for global deployment extend in most cases beyond 20 years, with coverage reaching no more than 80 per cent of the target population even in the best-case scenarios. These protracted timelines for much needed interventions against endemic infectious diseases come at a huge human cost and demand serious attention. Furthermore, the recent severe acute respiratory syndrome (SARS) outbreak, the continuing risk of a pandemic of severe avian influenza and the recent H1N1 pandemic have reinforced the need for a rapid global response to pandemics. For these reasons, the international community needs to be able to develop and deploy new vaccines more rapidly than has been the case in the past. In aiming to identify a blueprint for accelerated vaccine development, experts considered the current status of vaccine platforms in various parts of the world, reviewed some of the constraints to more rapid development and deployment of vaccines, and finally considered how some of these constraints might be addressed.

## 2. Current trends in vaccine development

While the world is affected today by a greater number of infectious diseases than ever before, many of which are zoonoses and mainly prevalent in poor countries, their infrequent occurrence in rich countries has led to a decline in investment, research infrastructure and expertise dedicated to infectious diseases in affluent, industrialized nations. Different perceptions of threat and public health priorities can thus be observed in three groups of countries: industrialized countries, innovative emerging economies and high-burden resource-poor settings. The attendees grappled with the relevance of these trends to the future of vaccine development.

### (a) Trends in industrialized countries

Although deaths from infectious disease still occur in wealthy countries, particularly in marginalized populations, improved public health measures have succeeded in reducing the toll from infectious diseases and, as a consequence, governments in rich nations are investing less in infectious diseases control and research. Investments in vaccine development have been very different for infectious diseases that affect both rich and poor countries, for example, human immunodeficiency virus (HIV) and human papillomavirus (HPV), compared with neglected tropical diseases (NTDs), which are exclusively endemic in low-income countries. Pharmaceutical companies are primarily focused on developing interventions for infectious diseases that affect industrialized nations, while investment in vaccines for NTDs has come largely from philanthropy, with most research and development (R&D) being undertaken by academic teams in alliance with a few product development partnerships (PDPs). While a huge investment in interventions for NTDs has already been made by charitable organizations such as the Bill and Melinda Gates Foundation, fear of lack of funding is reducing the size of the infectious diseases research community in some developed countries and weakening capacity for the exploratory and discovery activities that are key to identifying new interventions.

Significant anti-vaccination campaigns have taken root in many industrialized nations in spite of scientific findings refuting many unsubstantiated fears about vaccination. Attendees expressed concern that anti-vaccination bias in developed nations is not only having a serious negative impact on health campaigns in developing countries, but is also affecting the regulatory framework of the developed world, where the fear of litigation is compromising a balanced risk–benefit assessment of much needed interventions. As vaccine safety requirements become more demanding, the cost of vaccine development and exhaustive safety testing are dampening the incentive of the pharmaceutical industry to develop vaccines for diseases of populations with limited purchasing power.

### (b) Trends in innovative developing countries

In the past two decades, there has been an important change in the global economic and scientific landscape. The economies of China, India, South America, South Africa, Eastern Europe and the Middle East have risen in economic standing and many are investing substantially in state-of-the-art biomedical research infrastructures. These countries are placing substantial emphasis on the build-up of local scientific and technical expertise and are putting increasing resources into nurturing indigenous scientific talent. Pharmaceutical companies are increasingly seeking to manufacture vaccines in countries where the vaccines are to be marketed, leading to a number of productive partnerships between large multi-national companies like GlaxoSmithKline (GSK), Pfizer, Sanofi Pasteur and innovative developing country (IDC) governments. As a result, the number of production units for manufacturing drugs and vaccines and international research institutes operating effectively in IDC has expanded. The Developing Country Vaccine Manufacturers' Network (www.dcvmn.com), established in 2005, is increasingly engaged with supplying vaccines at affordable prices through UNICEF tenders to meet the health needs of the poor.

### (c) Trends in resource-poor developing countries

Sub-Saharan Africa continues to be the greatest concern as the prevalence of infectious diseases remains high and ability to pay for new health interventions remains low. In contrast to IDC countries, technology transfer to sub-Saharan Africa, with the exception of South Africa, remains low and there is little to no capital support for vaccine R&D. A lack of investment in vaccine and drug manufacturing is resulting in the dismantling of the few pre-existing manufacturing centres—a striking example being the loss of manufacturing capability that was set up in Nigeria for yellow fever vaccine. Given the positive examples of IDC countries, there is a major need to create and support regional centres of scientific and technical excellence in Africa. Programmes that support training of African scientists in partnership with international scientific centres and industry need to be encouraged through new and innovative means. Development agencies should be encouraged to invest in Africa to build capacity in infrastructure, with private and public investors as complementary pillars to that of international aid, and with African business players taking the lead in supporting technological advances that will serve the needs of their continent.

Attendees stressed that public health measures, including a number of successful vaccination programmes, have been greatly beneficial to both rich countries and the developing world; for example, vaccines against *Haemophilus influenzae* type b (Hib), pneumococcal and rotavirus infections. However, complacency, which dilutes the infrastructure for vaccine development, is very risky, especially at a time when increased international travel, evolution of drug resistance, climate change and the threat of bioterrorism make the age-old battle of humans against infectious disease more challenging. The re-emergence of killer diseases such as drug-resistant tuberculosis (MDR-TB) in Western Europe provides a warning that wealthy nations are not immune to the diseases of the poor. A versatile knowledge base and more frequent flow of information and technology transfer between industry and academia, and between disease endemic regions and knowledge-based economies, are essential, if public health measures are to fulfil their potential to improve the lives of populations globally.

Focusing on vaccine-based interventions, the experts set out to identify forces that would enable vaccine development to meet the demands of the twenty-first century. Three main agendas were reviewed: (i) achieving faster vaccine discovery and development; (ii) expediting the path of vaccine implementation in target populations; and (iii) securing increased financial aid for immunization programmes, supplemented with better advocacy to ensure strong public support for their implementation.

## 3. Vaccine discovery and development priorities

### (a) Accelerating experimental vaccinology

#### (i) Rapid testing of new platforms

**Figure 1.**
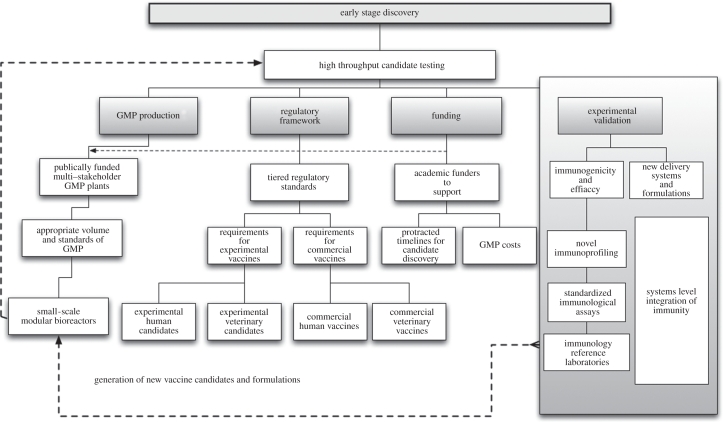
Strengthening early-stage vaccine discovery. The main components of early-stage vaccine candidate discovery and ways in which these could be strengthened to benefit accelerated proof-of-concept clinical testing.

**Figure 2.**
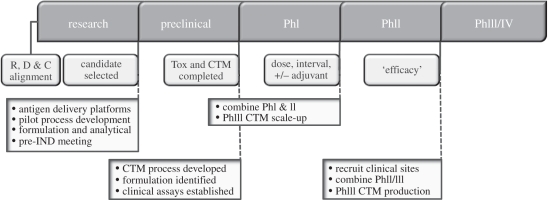
Accelerating the vaccine product development pathway. Ways in which candidate vaccines could be advanced to licensure within 8–10 years, if all players in the process are aligned from the outset. IND, investigational new drug application; CTM, clinical trial manufacturing. (Contribution of James Merson, Pfizer.)

The development of a successful human vaccine, from idea to licensure, is a complex process (figures 1 and 2) and is estimated to take over 15 years—a timeline judged by most to be too long. Most infectious diseases for which vaccines are needed urgently are far from being ‘easy targets’ and thus accelerated early-phase testing of vaccine candidates developed using a variety of different approaches is necessary to identify efficacious candidates. In the exploratory phase, more rapid and high-throughput testing of new vaccine antigens and delivery platforms is essential to establish early proof-of-concept. Usually, interest in these endeavours is driven by academic groups, but currently most lack access to the long-term investment in infrastructure required to undertake translational studies into humans. Conversely, industry requires a high-level of confidence in technologies and formulations required for product development if it is to permit early ‘at-risk’ investment. Lack of knowledge regarding protective immune responses, and/or how these can be induced in humans, condemn many difficult and scientifically neglected infectious diseases to the ‘too high risk’ bracket. Industry generally prefers tried and tested delivery platforms for new vaccines as these can be more easily aligned with established manufacturing capabilities and a commercial target product profile (TPP). This alignment leaves little to no room for innovative but unvalidated vaccine delivery technologies, such as formulations with improved thermal stability or needle-free administration, to enter the product development path. How then can the accelerated testing of new technologies be integrated with new product development? Two main approaches were proposed. Veterinary vaccines could lead the way in validating the performance of new technologies, establishing the amenability of the technology to manufacturing scale-up and to meeting regulatory standards. This would be supported by the lower cost of obtaining efficacy data, usually with microbial challenge studies, in host animal species. The validation and regulatory approval of novel technologies for veterinary interventions could reduce the risk of embedding the new technologies and manufacturing platforms into the development of human vaccines. Alternatively, human vaccine developers could be more proactive in testing innovative formulations and delivering technologies during the development of improved second-generation products. The introduction of new technologies in such a manner could lead into innovative delivery and formulation approaches reaching routine immunizations over the course of time, and overcome the current situation where some innovative technologies lapse because they are not taken up by the industry.

#### (ii) Infrastructure to support small-scale, high-throughput, vaccine manufacturing and clinical testing

The attendees suggested that new innovative ways of developing infrastructure to support small-scale, proof-of-concept vaccine development are of high priority. Similarly, manufacturing standards and production volumes should be tailored to assist early-phase vaccine development, making these endeavours more affordable and, most importantly, more high-throughput. The cost of good manufacturing practice (GMP) and associated quality control and quality assurance of experimental vaccines can be prohibitively high for academic groups and often similar standards are applied to small-scale manufacturing batches and manufacturing for market supply. The underlying high costs of GMP prevent important comparative testing of vaccine candidates in early-phase, proof-of-concept human clinical trials. An academic centre is fortunate if it secures sufficient funding to take even a single candidate vaccine through GMP manufacture and into a phase I clinical trial. It was stressed that suitable modifications of GMP standards could be identified that would not compromise safety, but could facilitate comparative testing of experimental vaccines. The use of single-product GMP production suites, or GMP labelling systems for experimental vaccines which are equivalent to labelling requirements for commercial vaccines were highlighted as two examples of requirements that could be adjusted for experimental vaccines to facilitate lower cost pilot GMP lot production. Utilization of new and more efficient vaccine production platforms, such as modular bioreactors and mobile clean rooms, could also help to meet this need in the future.

It was highlighted that in the field of veterinary medicine, the need is for ‘effective’ vaccines rather than ‘perfect’ vaccines. Veterinary vaccine regulatory requirements could be less stringent than for human vaccines and new GMP facilities could gain crucial experience in the manufacture of animal vaccines prior to proceeding with the more stringent production of human products. This approach could lead to the accelerated deployment of new innovative technologies and the establishment of new GMP facilities. This principle is already gaining some acceptance as multiple vaccines based on the new DNA-based vectors are now licensed for veterinary use, including 12 recombinant viral-vectored vaccines as well as four DNA plasmid vaccines (table 1). In contrast, in the human field, the first vectored vaccine, a chimeric flavivirus against Japanese encephalitis, is only just reaching regulatory approval.

**Table 1.** DNA and viral-vectored veterinary vaccines licensed for commercial use (adapted from Draper, S. J. & Heeney, J. L. 2010 *Nat. Rev. Microbiol.* **8**, 62–73).

recombinant vector	vaccine target/indication	target species	target antigen(s)	brand name	distributor
DNA	West Nile virus	horse	pre-membrane and envelope (preM-Env)	West Nile-Innovator	Fort Dodge Animal Health
DNA	melanoma	dogs	human tyrosinase	Oncept	Merial
DNA	infectious haematopoietic necrosis virus	salmon	glycoprotein	Apex-IHN	Novartis Animal Health
DNA	increase litter survival	swine	growth hormone releasing hormone	Life Tide SW5	VGX Animal Health, Inc.
attenuated Canarypox (ALVAC)	West Nile virus	horses	preM-Env	Recombitek Equine WNV	Merial
ALVAC (plus Tetanus Toxoid and Carbopol Adjuvant)	equine influenza virus	horses	haemagglutinin (HA) (Kentucky and Newmarket strains)	ProteqFlu-Te (Europe) Recombitek (USA)	Merial
ALVAC	rabies	cats	glycoprotein G (gG)	Purevax Feline Rabies	Merial
ALVAC	feline leukaemia virus	cats	Env, Gag/Pol	Purevax FeLV	Merial
ALVAC	canine distemper virus	dogs	HA and fusion antigen (F)	RECOMBITEK rDistemper	Merial
ALVAC	canine distemper virus	ferrets	HA and F	Purevax Ferret Distemper	Merial
Fowlpox virus (FPV)	avian influenza virus and FPV	poultry	H5 HA	Trovac AI H5	Merial
FPV	Newcastle disease virus (NDV) and FPV	poultry	haemagglutinin-neuraminidase (HN) and F	Vectormune FP-N	Biomune
Vaccinia	rabies virus	wildlife	gG	Raboral	Merial
Newcastle disease virus (LaSota strain)	avian influenza virus and NDV	poultry	H5 HA	NewH5	Avimex
Flavivirus YFV-17D (live chimeric virus)	West Nile virus	horses	preM-Env of WNV in yellow fever virus (YFV)-17D backbone	PreveNile	Intervet
Turkey herpesvirus (HVT) (live chimeric virus)	infectious bursal disease virus (IBDV) and Marek's disease virus (MDV)	poultry	viral protein 2 (VP2) of IBDV in HVT backbone	Vaxxitek HVT + IBD	Merial

In order to facilitate vaccine discovery, the development of more academic vaccine centres should be encouraged, which allow for vaccine development to take place in fruitful, multi-disciplinary environments. Funders of academic groups working on translational research need to appreciate that the timelines for vaccine development will exceed those of traditional research grants in order to provide continued incentive for academics to work in this area. Calls were made for funders to invest in new technologies as well as in the next generation of scientists who will pursue careers in vaccine development and who will be able to apply recent advances in immune profiling and systems biology to the development of innovative new vaccines against the most elusive diseases.

#### (iii) Multi-stakeholder support for the infrastructure needed for vaccine development

Vaccine development requires the input of a hugely diverse skill set, and it was suggested that a much better integrated, multi-stakeholder and shared funding approach could help to accelerate early-phase vaccine development. Pharmaceutical companies have invested heavily in recent years to provide infrastructure that can cater for twenty-first century needs relating to antimicrobials, therapeutics, vaccines and diagnostics. In developed countries, designated facilities have also been established through public funds to address the needs of biodefence and the threat from newly emerging infectious and zoonotic diseases. It was suggested that similar multi-stakeholder, publicly funded, GMP facilities could be established to provide a cost-effective means to develop multiple, experimental vaccine candidates. These could potentially cater for a wide range of new generation vaccine delivery technologies and, importantly, could provide capacity for very rapid emergency vaccine manufacture in response to disease outbreaks—a so-called model of ‘PERManEnt facility’ (*P*andemic *E*mergency *R*esponse *Man*ufacturing *Ent*erprise) was proposed. This type of facility would aim to shorten the current timelines for new vaccine development in the event of a new SARS-like outbreak from months to weeks. However, it was noted that issues of liability could deter the participation of industry in such multi-stakeholder GMP ventures.

#### (iv) Access to vaccine adjuvants—a global ‘crisis’?

The global ‘crisis’ regarding access to promising vaccine adjuvants was discussed. A significant difficulty for vaccine research in academia has been the lack of access to many of the promising adjuvants, developed by companies that have been unwilling to share these with external investigators. Greater ease of access to such adjuvants could significantly accelerate experimental vaccinology and expedite identification of preferred adjuvants for particular vaccines. Lack of access has prevented comparative testing of many adjuvant formulations both in pre-clinical and clinical studies, and has had a particularly adverse effect on vaccine development for diseases for which there is limited commercial interest and for which very strong immune responses are required for protection. In an attempt to remedy this problem, some non-profit organizations, with support from major funding agencies, have established a new Global Adjuvant Development Initiative (GADI) laboratory under the guidance of the World Health Organization (WHO), located at the University of Lausanne. GADI is supporting mechanisms to make adjuvants available to public-sector vaccine developers. It was stressed by delegates from industry that liability issues can prohibit the sharing of adjuvants used for commercial vaccines, but that some adjuvants which are in the R&D pipeline could be shared with academic and public-sector partners.

### (b) Accelerating the product development path

While doubts were expressed as to whether it is possible to expedite early-phase candidate vaccine testing for difficult diseases, it was agreed that there is more scope for progress with regard to accelerated product development pathways. Early alignment of key players in the process is essential, and this can help to establish a clear line of sight from product development to vaccine registration. Strides should be made to establish the TPP, essentially defining the ‘label on the vial’ from the outset, start pilot process development at the same time as R&D and instigate a process of small, rapid, parallel clinical trials to ascertain safety and efficacy in the target population, ideally also identifying a bioassay of vaccine efficacy. In order to expedite vaccine licensure, clinical testing should consist of ‘rolling’ phase I/II trials leading into a phase III trial in the minimum number of countries with all bioassays performed in a single reference laboratory. The clinical testing of Prevnar-13 in The Netherlands was highlighted as a best-practice example. If all these processes are aligned, candidate vaccines can be advanced to registration in 8–10 years (figure 2). It was noted that acceleration of the pathways that lead to product licensure will most probably require a larger set of post-licensure studies (e.g. of performance in special populations, extended safety, impact on other immunizations); however, this was viewed as a favourable strategy in the context of benefits arising from accelerated vaccine deployment.

## 4. Implementation priorities

### (a) The science of implementation: identifying best-practice for taking vaccines from licensure to deployment

**Figure 3.**
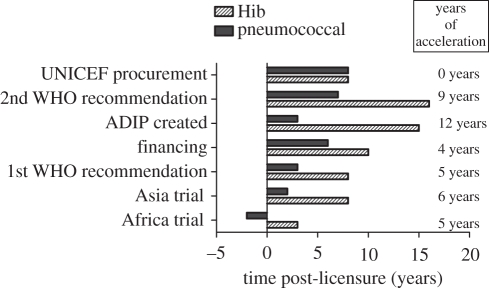
Accelerating vaccine delivery. Comparison of the timeline for the processes involved in the implementation of pneumococcal vaccine versus the implementation of Hib vaccine. ADIP, accelerated development and introduction plan. (Contribution of Orin Levine, Johns Hopkins Bloomberg School of Public Health.)

Once licensed, vaccines still face an uphill struggle to ensure wide-scale deployment in the communities where the vaccines have the greatest potential to make an impact. It seems likely that the deployment of pneumococcal conjugate vaccines in developing countries will be faster than that achieved for the Hib conjugate vaccine, in part as a result of lessons learnt during the course of the activities of the Hib initiatives and those of the pneumococcal accelerated development and introduction of priority new vaccines plan (ADIP; figure 3). These two immunization programmes provide a striking example of how much could be gained by improving the process of vaccine implementation. The ‘science of vaccine implementation’ still remains in its infancy, although there are success stories at national level such as the introduction of the HPV vaccine for teenage girls in the UK in 2008/2009 with greater than 80 per cent coverage achieved in the first year. Positive examples and best practice at national and international levels could be used to design a guiding framework for vaccine deployment with maximal coverage and minimal time lag between licensure and widespread deployment.

Better understanding of the data required to guide vaccine implementation and the development of ‘checklists’ to guide decision-making at various levels in the implementation of vaccination programmes were considered of paramount importance for efficient vaccine introduction. Dedicated teams are required to work on the development of these processes and guidelines. As part of the implementation preparedness, disease endemic countries need to establish a strong ‘pull’ factor that ensures demand for a vaccine. This requires governments of these countries to understand the burden of disease, be engaged in implementation plans, contribute to vaccine procurement and advocate their use. This process will require long-term collaborations to be established between international public health agencies, national health authorities and industry.

## 5. Regulatory reform

Regulatory reforms were viewed as important ways to achieve faster clinical testing and licensure of new, innovative vaccines. Discussion focused on whether a universal regulatory framework or a case-specific assessment of risks and benefits linked to each medical product would be better placed to support health interventions in the twenty-first century. It was noted that maintaining consistent global standards with regard to risk–benefit analysis is essential. However, it was stressed that no universal interpretation of such an analysis can apply as disease epidemiology, morbidity and mortality vary so widely with geographical location.

The development of a methodological framework based on consistent and universally acceptable standards of risk–benefit analysis would support a regulatory assessment that takes into consideration how a specific preventive intervention impacts on the disease and health pattern in a particular region. A legal framework that offers flexibility for a case-by-case assessment on a benefit–risk basis, with rigorously maintained standards, was seen as the best way to maintain the timely and rapid licensure of new vaccines. It was agreed that weak, inflexible regulation would hinder, rather than accelerate, the progression of much needed vaccines to market, as well as risk undermining public and industrial confidence in the regulatory system. The need to structure regulatory standards in tiers for human versus veterinary vaccines and experimental versus commercial applications was identified as a timely reform that would transform the regulatory landscape (figure 1).

In the case of veterinary vaccines, comparisons were drawn between developing countries, where the regulatory infrastructure remains weak, and developed countries, where regulation is strong and detailed. Examples were cited from the European Union, where manufacturing standards can be considered as unnecessarily high for some veterinary vaccines, making the production of these vaccines fairly expensive. Many European farmers are unwilling to pay such high prices, leading to potential loss of vaccine demand or market ‘pull’ factors. This was contrasted to developing countries where, for example, the Maasai in East Africa are willing to pay relatively high prices for cattle vaccines (up to US$4), owing to their understanding that healthy livestock are a pathway out of poverty. Striking the right regulatory balance in the veterinary field will be essential to the future deployment and widespread use of accessible and affordable veterinary vaccines to the millions in developing countries for whom livestock are a lifeline.

## 6. Increased financial and public support for immunization programmes

### (a) Innovative partnerships and financing

Given the need to combat highly complex infectious diseases and the unequal distribution of resources to meet these challenges across geographical locations, support for initiatives that increase the diversity of technical, organizational and institutional arrangements in which multi-disciplinary scientific research is conducted was considered of paramount importance. Identification of positive examples and dissemination of knowledge about the value obtained from successful industry–academia–public-sector partnerships can help in the design of new collective institutions or partnerships. The attendees emphasized that policymakers and funders need to find new ways to create the conditions needed for entrepreneurial, multilateral enterprises and then to initiate and fund them. In Europe, the Wellcome Trust has expanded its funding portfolio to include joint ventures between industry, academic groups and PDPs. The establishment of the MSD-Wellcome Trust Hilleman Laboratories in India in 2009 is a prime example of a research charity and a pharmaceutical company engaging in a joint venture with equally shared funding and decision-making rights to establish a research centre with a focus on developing vaccines for diseases of poverty. This partnership combines the ingenuity of academic research with the know-how of industry, aiming to integrate the best of both in setting up a research centre that operates like a business with product-focused goals, but with a not-for-profit financial model. This is a an ongoing experiment which, if successful, may encourage others to follow this direction and help to address the critical gap that results in promising experimental approaches not getting translated into health interventions because of low profitability. Another model of multiparty collaborative networks is the TRANSVAC infrastructure project (www.transvac.org), funded under the European Commission's 7th Framework Programme and coordinated by the European Vaccine Initiative (EVI), set up to create a cluster of knowledge and research infrastructure for the respective benefit of collaborative partners to have access to adjuvants, animal models, standardized reagents and assays, as well as microarray analysis. The experts recognized that there is a need for various stakeholders to commit knowledge, expertise and funds to ensure that these initiatives prosper.

### (b) Improved priority setting and advocacy

Establishing ‘end-user priorities’ was seen as one of the most critical issues needed to ensure that the vaccine development pipeline meets true needs in the field, and this prioritization strategy was seen as an important component of the best-practice implementation programme. There was a consensus that communication and engagement with governments and healthcare workers in disease endemic regions should start as early as possible in vaccine development programmes. A multi-level model of stakeholder engagement needs to be developed, which takes into account the different needs of various target groups as well as any epidemiological changes that may take place during the 10–15 years of vaccine development. To better understand the needs of the end-users, emphasis was put on community engagement and the use of culturally appropriate informed consent for clinical studies. The demand for Halal certification to gain consumer confidence in the Middle East was used as an example to highlight the importance of an early appreciation of end-user expectations.

Improved understanding between governments of disease endemic regions, the WHO, the Global Alliance for Vaccines and Immunization (GAVI) and industry was considered essential to guide implementation decisions for new vaccines. The possibility of allowing countries to lodge applications for vaccines before pre-qualification was proposed as a means to accelerate vaccine availability. It was also emphasized that GAVI needs to engage with governments of developing countries to ensure that their national health budgets have provisions to support vaccination after GAVI subsidies end. This will help to establish a long-term ‘pull’ mechanism for new interventions in endemic regions.

Similarly, the importance of establishing the driving forces within the commercial market was also stressed. The role of farmers and pet owners as primary customers for veterinary vaccines was contrasted to human vaccines, whereby purchasing occurs at the level of national health systems and where advocacy and political will for targeting a specific public health need to underpin the ‘pull’ factor for a specific intervention.

Endeavours to develop life-saving vaccines must be matched by clear and accessible information about the value of vaccination. Governments and private funders must be provided with evidence-based information about the need for investment in vaccine development and support for immunization programmes, while the public must be provided with clear and accessible information about the value of vaccination as a public health intervention. A greater role was seen for national academies of sciences and learned societies in promoting scientific-based support for vaccination programmes.

For communication strategies to have an impact, opinions and recommendations need to come from experts who are independent of profit-making interest groups and of government influence. One proposal was put forward to set up independent vaccine information institutes as information dissemination centres committed to the improvement of public knowledge about vaccines. No matter how strong the science may be and how uniform the expert consensus, the general public will remain sensitive to alarms raised by anti-vaccination movements, and will be swayed by the opinions of friends, actions of their peers and the media. This needs to be accepted and responded to by teaming up independent and informed advisors with parents' groups, opinion leaders and media experts who can address any real or unfounded fears and explain the risks of vaccine apathy as well as vaccine use, adopting the spectrum of modern communication channels to which the younger generation is most amenable.

## 7. Conclusion

The meeting concluded with a review of the issues that had been identified during the course of the discussion as challenges to the accelerated development of new vaccines (box 1), and with the identification of some of the ways in which these challenges could be met (box 2).

Box 1. **Challenges to the acceleration of vaccine development and deployment.**

— A growing number of academic centres are becoming involved with experimental vaccinology. However, funders of academic teams are not aware of the protracted timelines and costs required for vaccine discovery and clinical development.
— Fragmentation of immunological research into domains of infectious disease and cancers of humans and animals has thwarted the synergy that could be obtained by undertaking a more combined tactical approach.
— The costs of GMP and toxicology testing are prohibitively high for new proof-of-concept vaccines developed by academic groups.
— Uptake of new innovative vaccine delivery platforms by industry remains low. Little technology and knowledge transfer occur at the early stages of vaccine development.
— Cross-fertilization of ideas and technologies between industry and academia remains weak.
— The ‘science of vaccine implementation’ is under-developed and long gaps between registration and effective implementation are responsible for the loss of many human lives.
— The TPP of a vaccine should be developed early and tailored to the need(s) of the target population.
— The communication between different vaccine stakeholders during the course of vaccine development and implementation needs improving.
— Emerging economies like China, India, Brazil, etc. are investing more in their health-based capabilities both in terms of resources and expertise, but sub-Saharan African countries are lagging behind.
— Regulatory frameworks are not flexible enough to facilitate accelerated testing and introduction of innovative technologies.
— Financing remains an issue for NTDs. While there is an increase in financial support from philanthropic donors, public funding for vaccines against these diseases has declined both in Europe and in the USA.
— The influence of anti-vaccination lobbies threatens to seriously undermine public confidence in immunization programmes and vaccines.

Box 2. **Recommendations on how to tackle some of the challenges that are impeding the development and deployment of new vaccines.**

— Best practices that could shorten the path of vaccine development from 15–20 years to 10–15 years need to be identified, validated and disseminated.
— The discovery phase for experimental vaccinology should be strengthened by setting up publically supported, multi-stakeholder GMP and toxicology facilities to support ‘experimental vaccines’. Introduction of modular/self-contained manufacturing bioreactors could enable faster throughput of vaccine candidates for proof-of-concept clinical trials.
— A more rational approach to vaccine design should involve support for more multi-disciplinary investigations of the immunological and systems biological basis of disease and protective immunity.
— Veterinary vaccines and second-generation products should lead the way for validating new and innovative enabling technologies.
— Regulatory agencies should be more flexible and innovative in setting up a system of tiered regulatory standards tailored to veterinary and human vaccines, which differentiates between experimental and commercial applications.
— Regional capacity to support vaccine clinical testing should be strengthened.
— A best-practice method for managing vaccine implementation programmes to support a rapid transition from vaccine licensure to national immunization programmes should be developed.
— Improved communication and collaborations with the governments of high-burden disease countries are needed to ensure that the needs of end-users are understood and met.
— National Academies of Sciences could help in producing the information needed to counter the efforts of the anti-vaccination lobbies.
— Establishing independent vaccine information institutes, at the national level, to complement WHO efforts and deliver online, accurate, publically accessible information related to vaccines should be considered.

